# Lasofoxifene as a potential treatment for therapy-resistant ER-positive metastatic breast cancer

**DOI:** 10.1186/s13058-021-01431-w

**Published:** 2021-05-12

**Authors:** Muriel Lainé, Sean W. Fanning, Ya-Fang Chang, Bradley Green, Marianne E. Greene, Barry Komm, Justyna D. Kurleto, Linda Phung, Geoffrey L. Greene

**Affiliations:** 1 The Ben May Department for Cancer Research, The University of Chicago, 929 East 57th Street, GCIS W421C, Chicago, IL 60637 USA; 2 Department of Cancer Biology, Loyola University Chicago, Maywood, IL USA; 3 Komm-Sandin Pharma Consulting, Newtown Square, PA USA

**Keywords:** Breast cancer, Endocrine resistant, Fulvestrant, Lasofoxifene, Selective estrogen receptor modulator

## Abstract

**Background:**

Endocrine therapy remains the mainstay of treatment for estrogen receptor-positive (ER+) breast cancer. Constitutively active mutations in the ligand binding domain of ERα render tumors resistant to endocrine agents. Breast cancers with the two most common ERα mutations, Y537S and D538G, have low sensitivity to fulvestrant inhibition, a typical second-line endocrine therapy. Lasofoxifene is a selective estrogen receptor modulator with benefits on bone health and breast cancer prevention potential. This study investigated the anti-tumor activity of lasofoxifene in breast cancer xenografts expressing Y537S and D538G ERα mutants. The combination of lasofoxifene with palbociclib, a CDK4/6 inhibitor, was also evaluated.

**Methods:**

Luciferase-GFP tagged MCF7 cells bearing wild-type, Y537S, or D538G ERα were injected into the mammary ducts of NSG mice (MIND model), which were subsequently treated with lasofoxifene or fulvestrant as single agents or in combination with palbociclib. Tumor growth and metastasis were monitored with in vivo and ex vivo luminescence imaging, terminal tumor weight measurements, and histological analysis.

**Results:**

As a monotherapy, lasofoxifene was more effective than fulvestrant at inhibiting primary tumor growth and reducing metastases. Adding palbociclib improved the effectiveness of both lasofoxifene and fulvestrant for tumor suppression and metastasis prevention at four distal sites (lung, liver, bone, and brain), with the combination of lasofoxifene/palbociclib being generally more potent than that of fulvestrant/palbociclib. X-ray crystallography of the ERα ligand binding domain (LBD) shows that lasofoxifene stabilizes an antagonist conformation of both wild-type and Y537S LBD. The ability of lasofoxifene to promote an antagonist conformation of Y537S, combined with its long half-life and bioavailability, likely contributes to the observed potent inhibition of primary tumor growth and metastasis of MCF7 Y537S cells.

**Conclusions:**

We report for the first time the anti-tumor activity of lasofoxifene in mouse models of endocrine therapy-resistant breast cancer. The results demonstrate the potential of using lasofoxifene as an effective therapy for women with advanced or metastatic ER+ breast cancers expressing the most common constitutively active ERα mutations.

**Supplementary Information:**

The online version contains supplementary material available at 10.1186/s13058-021-01431-w.

## Introduction

Breast cancer is the most common cause of cancer mortality in women worldwide [1]. Approximately 80% of breast cancers are estrogen receptor positive (ER+) [2]. Evidence suggests that estrogen receptor alpha (ERα, encoded by *ESR1*), a member of the nuclear receptor family of transcription factors, is involved in the initiation and progression of ER+ breast tumors [3]. Estradiol (E2) binding to the ER causes receptor dimerization to its active form for coactivator binding, leading to enhanced proliferation and survival of breast epithelial cells through the transcriptional modulation of genes [3, 4].

Endocrine therapy that inhibits ERα activity remains the mainstay of treatment for ER+ breast cancer. Tamoxifen, a selective estrogen receptor modulator (SERM), acts as a partial antagonist for ERα, and aromatase inhibitors (AIs) inhibit estrogen production [3]. However, a number of patients either are de novo resistant to these therapies [5] or become resistant after prolonged exposure to these drugs [6], and experience cancer recurrence in the 5 to 20 years following treatment completion [7]. Fulvestrant (FUL), the only approved selective estrogen receptor down regulator (SERD), has been shown to be effective in treating endocrine therapy-resistant tumors [8], but the challenges of drug resistance remain even for FUL [9].

Several mechanisms have been suggested for endocrine-therapy resistance [4, 6]. One important mechanism involves acquired *ESR1* mutations under the selective pressure of endocrine therapy treatments, especially aromatase inhibitors. The *ESR1* mutations are detected almost exclusively in the ligand binding domain (LBD), which includes the major transcriptional activating function-2 (AF2) [3]. In patients with metastatic ER+ breast cancer, these mutations have been observed at a frequency of 10–40%, but are rarely detected in primary tumors [10–12]. The two most common *ESR1* mutations are Y537S and D538G, located at the N-terminus of helix 12 (H12), a key structural regulator of coactivator recruitment in the LBD of ERα [10–13]. The conformational changes in H12 caused by these mutations give rise to a constitutively active receptor that can interact with coactivators, independent of ligand, and has reduced affinity for agonists and antagonists, thereby conferring reduced sensitivity of the mutants to endocrine drugs such as tamoxifen or FUL [10–14]. Additionally, allele-specific neomorphic properties found in these mutants could also contribute to cancer metastasis [15]. These limitations prompted the search for new treatment strategies that would be effective against breast cancers expressing known ERα mutants, including a SERM that might also alleviate postmenopausal symptoms related to estrogen loss, while inhibiting breast cancer progression. Raloxifene is the only SERM other than tamoxifen currently approved for reducing invasive breast cancer risk in postmenopausal women [16]. Bazedoxifene, a third-generation SERM, has shown potential anti-tumor effects in animal models with therapy-resistant breast cancer [17, 18].

Lasofoxifene (LAS), also a third-generation SERM, was developed to treat postmenopausal vaginal atrophy and osteoporosis [19] and approved but not used in Europe for osteoporosis treatment in postmenopausal women at increased risk of fracture [20]. In the Postmenopausal Evaluation and Risk-Reduction with Lasofoxifene (PEARL) trial, 5 years of LAS was associated with reduced risk of 79% for total breast cancers and 83% for invasive ER+ breast cancer and had beneficial effects on vertebral and non-vertebral fractures, coronary heart disease events, and stroke [21]. A network meta-analysis of randomized controlled trials on breast cancer chemoprevention agents showed that similar to raloxifene, LAS elicited a better benefit-risk profile than tamoxifen and AIs [22]. However, LAS has not been tested as a therapeutic option for progressive breast cancer. A recent cell-based study showed that the antagonist activity of LAS on ER+ breast cancer cells was not affected by the expression level of activating ERα mutants relative to wild-type (WT) ERα, a property not observed for other agents tested, including tamoxifen, bazedoxifene, raloxifene, and FUL [23].

The objective of these studies was to investigate the potential benefit of LAS on endocrine-resistant ER+ metastatic breast cancer (MBC) in human-derived xenograft models harboring Y537S and D538G *ESR1* mutations, the most commonly observed ERα mutations. In addition, effectiveness on tumor inhibition by LAS combined with palbociclib (PAL), a CDK4/6 inhibitor that blocks cell-cycle progression and has been shown to enhance the efficacy of endocrine agents [24, 25], was evaluated. Notably, LAS alone or in combination with PAL was an effective inhibitor of tumor growth in the MCF7 Y537S ERα+ MBC xenograft model. Additionally, the LAS/PAL combinations were notably more effective than the FUL/PAL combinations at inhibiting tumor growth and metastasis to the lung, liver, brain, and long bones. Structural analyses showed that LAS stabilizes an antagonist conformation of both WT and Y537S ERα LBDs.

## Materials and methods

### Cell lines

MCF7 breast cancer cells bearing WT and mutant (Y537S and D538G) ERα were kindly provided by Dr. Ben Park of the Sidney Kimmel Cancer Center at Johns Hopkins University [26]. The mutant cell lines were heterozygous, with both WT and mutant ERα expressed. MCF7 cells were transduced with a lentivirus vector (pFU-Luc2-eGFP) encoding a luciferase and green fluorescent protein (GFP) fusion gene driven by a ubiquitin promoter at a MOI = 5 in suspension and then plated [27]. Cells were cultured in DMEM containing 5% FBS and genotyped prior to injection in mice. DNA was extracted with DNeasy Blood and Tissue Kit (Qiagen Cat#69504) and the presence of the ERα mutations was verified by sequencing with CCCCTTCTAGGGATTTCAGC as the sequencing primer.

### Breast cancer models

Mouse studies were performed in compliance with an approved Institutional Animal Care and Use Committee protocol at the University of Chicago. To better mimic the progression of ductal lesions to invasive disease, a mammary intraductal (MIND) mouse model [28, 29] was used. NSG (NOD.Cg-Prkdc^scid^ Il2rg^tm1Wjl^/SzJ) mice (Jackson Laboratories) were anesthetized by intraperitoneal injections with a ketamine/xylazine (100/5 mg/kg) mixture in PBS prior to cancer cell injections. Single-cell suspensions of 250,000 WT or mutant MCF7 cells were injected in the mammary ducts of inguinal glands as previously described [28, 29]. Tumor cell growth in situ was followed once weekly or biweekly after injection via luminescence imaging with a Xenogen IVIS 200 instrument in the Integrated Small Animal Imaging Research Resource at the University of Chicago. Mice were injected with 100 μL of 0.1 M luciferin in PBS (Perkin Elmer XenoLight Cat#122799) prior to imaging. Effects were evaluated in the models expressing WT, Y537S, and D538G ERα for monotherapies, and in the model expressing Y537S ERα for combination therapies.

### Treatment

Treatment was initiated 2 to 3 weeks (dose-response study) or at day 20 (combination study) after cancer cell injections. For the dose-response study, LAS at 1, 5, or 10 mg/kg in 100 μL of PBS containing 15% PEG 400, or vehicle, was administered 5 days/week via subcutaneous (s.c.) injection, while FUL 5 mg/mouse (Med Chem Express Cat#HY-13636) in 100 μl of mineral oil was injected s.c. once per week. For the combination study, PAL (Med Chem Express Cat#HY-50567) at 35 or 70 mg/kg in 100 μL of 50 mM sodium lactate (pH4) was administered via oral gavage 5 days/week either alone or in combination with LAS or FUL. Mice were sacrificed 70 days after treatment initiation (dose-response study) or 82 days after cancer cell injection (combination study), and mammary gland tumors were weighed. Metastases were evaluated with ex vivo imaging of excised long bones, brains, lungs, and livers and quantified by luciferase activity for the combination study.

### Histological analysis

Tissues were harvested and fixed in formalin. Hematoxylin and eosin (H&E) staining was performed for livers and long bones. Immunohistochemical (IHC) staining was performed for lungs with human cytokeratin AE1/AE3 (dose-response study) or anti-luciferase antibody (combination study), and for long bones with anti-luciferase antibody. Histological analysis was performed by the Human Tissue Resource Center (HRTC) at the University of Chicago. Primary antibody for human cytokeratin AE1/AE3 (Biocare Medical Cat#CM011A) or antiluciferase antibody (abcam #181640) was used at 1/2000 dilution to visualize tumor cells. IHC and H&E stained sections were scanned on a CRi Pannoramic Scan whole slide scanner. A Nikon Eclipse Ti2 microscope with a ×10 objective was used to obtain high-resolution images. Percent area of liver metastasis (the area of tumor cells normalized to the total liver area) was analyzed using the ImageJ-FIJI software. Lung and bone metastases were analyzed using the NSI-Elements software from Nikon. Results were plotted as histogram +/− SEM or using a boxplot.

### Crystallography

The WT and Y537S ERα LBDs with all solvent-exposed cysteines mutated to serines were expressed in *Escherichia coli* BL21(DE3) and purified using Ni-NTA resin and size exclusion chromatography, as previously described [30]. Peak fractions corresponding to dimeric LBD were pooled and concentrated to 10 mg/mL. The products were then flash frozen and kept at −80°C. For crystallization, proteins were thawed on ice and LAS was added at 1 mM for up to 3 h on ice. Hanging drop vapor diffusion was used to grow protein crystals. Each complex was crystallized in either PEG 3350 or PEG 8000 at pH 6–8 with 200 mM MgCl_2_. X-ray data sets were collected on the SBC 19-BM beamline at the Advanced Photon Source Argonnne National Laboratory. Data were scaled, merged, and integrated using HKL-3000. Structures were solved at 1.8 Å for WT ERα LBD complex and 2.1 Å for Y537S ERα LBD complex using molecular replacement and manual coordinate editing, and refined using Phenix and Coot. All coordinates and structure factors were deposited in the Protein Data Bank (PDB) with accession codes 6VJD for the WT LBD/LAS complex and 6VGH for the Y537S LBD/LAS complex. Supplementary Table S1 shows x-ray crystallographic data collection and refinement statistics. Supplementary Figure S1 shows 2mFo-DFc difference maps for representative LAS ligands in the ERα LBD binding pocket for the WT and Y537S structures.

### ER binding assays

The purified ERα LBDs of 5 nM were incubated with 10 nM [^3^H]-E2 with varying concentrations (0.1 nM to 10 μM) of competitors (LAS and 4-hydroxytamoxifen [4-OHT]) in binding buffer (10 mM Tris pH 7.6, 300 mM NaCl, 5 mM EDTA, 10% glycerol, 1 mM DTT). After a 1-h incubation on ice, 50 μL of each reaction was loaded onto a CPG column at 4 °C in triplicate and incubated for 5 min. The columns were then washed with approximately 10 mL wash buffer (10 mM Tris pH 7.4, 400 mM NaCl) to remove unbound ligands. Remaining [^3^H]-E2 was eluted with 1 mL ethanol and counted in a liquid scintillation counter. Data were analyzed as described previously [13, 31].

### Statistical analysis

GraphPad Prism 7 software was used to analyze data and create graphs and boxplots. *P*-values were determined using unpaired, two-tailed *t*-test, Fisher’s exact test, or one-way ANOVA, with *p* ≤ 0.05 considered statistically significant.

## Results

### Tumor growth with LAS monotherapy

**Fig. 1:**
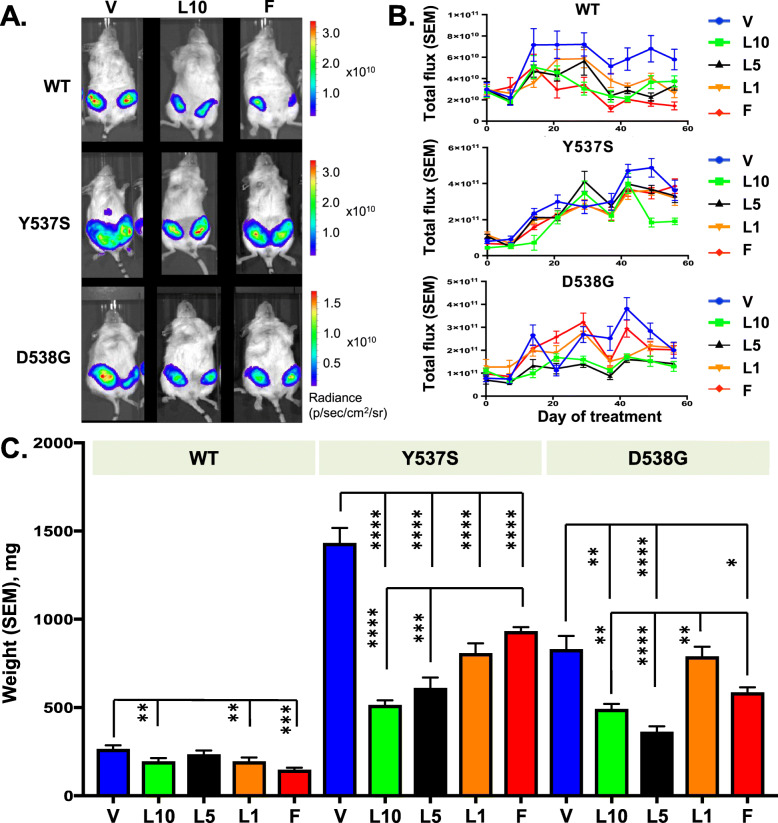
Effect of lasofoxifene on the progression of primary tumors expressing WT and mutant ERα. **a** Representative in vivo luminescence images of mice bearing tumors expressing MCF7 WT, Y537S, and D538G ERα at day 56 after treatment initiation. **b** Total photon flux of luminescence signals measured by in vivo imaging for tumors expressing MCF7 WT, Y537S, and D538G ERα over time (*n* = 8–10). **c** Average weight of mammary glands at sacrifice (*n* = 8–10 glands). *p < 0.05, **p < 0.005, ***p < 0.0005, ****p < 0.0001 by Student’s *t*-test. F, fulvestrant; L1, L5, and L10, lasofoxifene at 1, 5, and 10 mg/kg; SEM, standard error of the mean; V, vehicle

Tumors derived from mutant Y537S and D538G cells grew faster than tumors from WT MCF7 cells. In vivo real-time luminescence imaging showed that LAS alone (5 and 10 mg/kg) reduced tumor mass compared with vehicle control in xenograft tumors expressing WT, Y537S, and D538G ERα, similar to FUL (Fig. 1a and Supplementary Figure S2). For the two mutants, the total photon flux quantification of luminescence signal over time demonstrated that LAS was more effective than FUL at inhibiting tumor growth, with the highest dose of LAS (10 mg/kg) eliciting a superior inhibitory effect versus FUL (Fig. 1b). Based on observed radiance alone, potential tumor shrinkage with vehicle control towards day 56 after treatment initiation was observed for Y537S and D538G tumors (Fig. 1b), which was likely an artifact as this phenomenon was not observed in later experiments.

Data on final primary tumor weight at sacrifice correlated with the in vivo luminescence imaging results, with a more obvious and statistically robust dose effect for LAS, especially for the Y537S mutant. Significantly lower terminal tumor weights were observed for FUL and LAS relative to vehicle in the three mouse models (Fig. 1c). Reduction in tumor weight with 5 and 10 mg/kg of LAS was approximately 60% for Y537S and 50% for D538G. Final tumor weights were much lower in mice injected with MCF7 cells expressing WT ERα compared with the other two MCF7 variants, reflecting the slower growth of WT versus mutant tumors, as expected. LAS 5 or 10 mg/kg resulted in significantly lower tumor weights for both ERα mutants versus FUL. However, FUL appeared to be more effective than LAS at inhibiting WT tumor.

### Metastasis with LAS monotherapy

**Fig. 2:**
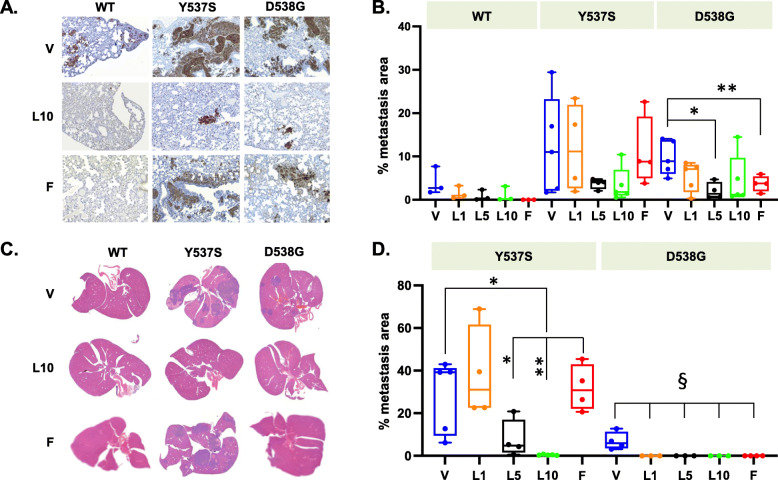
Lasofoxifene reduced lung and liver metastases. **a** Representative areas of IHC staining with human cytokeratin AE1/AE3 showing lung metastases (brown). **b** Percent of metastasis area normalized to total lung area (*n* = 4–5). *p < 0.05, **p < 0.005 by Student *t*-test. **c** Representative H&E staining of livers. **d** Percent metastasis area normalized to total liver area (*n* = 3–5). *p < 0.05, **p < 0.005 by Student *t*-test for Y537S; ^§^*p* = 0.05 by Fisher’s exact test for D538G. F, fulvestrant; L1, L5, and L10, lasofoxifene at 1, 5, and 10 mg/kg; V, vehicle

Histological analysis revealed metastases of the primary tumor to the lung and the liver in vehicle-treated mice, with the total area of lung metastases considerably larger for Y537S and D538G than for WT (Fig. 2). LAS at 10 mg/kg decreased lung metastases in all three mouse models as shown by IHC staining (Fig. 2a). In WT tumors, LAS at all tested doses reduced lung metastases to a level comparable to that by FUL (average percent area of metastasis: 4% by vehicle, 0.9–1.5 % by LAS, and 1% by FUL, *p* = NS; Fig. 2b). For both mutant tumors, the inhibitory effect of LAS appeared dose dependent, with the two higher doses almost completely blocking metastasis, although a significant difference versus vehicle was only observed for 5 mg/kg LAS in D538G mice. FUL versus vehicle completely blocked lung metastasis of WT tumors and significantly blocked lung metastasis of D538G but not Y537S tumors.

Similarly, H&E staining showed that LAS inhibited metastasis to the liver for both mutants, while FUL only had an effect on D538G (Fig. 2c). Quantification of metastatic coverage confirmed that LAS at 5 and 10 mg/kg was significantly more effective at reducing liver metastases of Y537S mutant cells compared with vehicle and FUL (Fig. 2d). The D538G tumors were less metastatic (with smaller metastases) than the Y537S tumors in the liver, and no liver metastases were observed in D538G mice treated with LAS or FUL (Fig. 2d).

### Effect of combination therapies on tumor growth

**Fig. 3:**
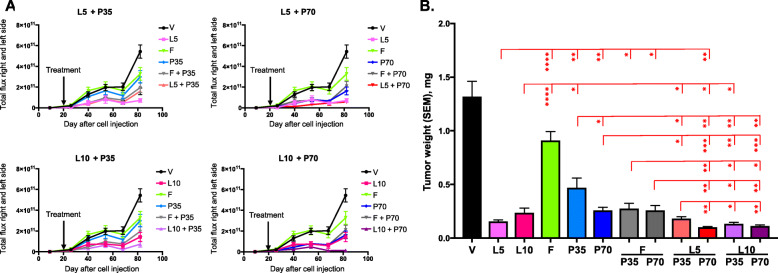
Effect of combination therapies on primary tumor growth in the Y537S ERα mutant model. **a** Total photon flux of luminescence signals measured by in vivo imaging over time for different dose combinations of lasofoxifene and palbociclib. **b** Tumor weight at sacrifice (*n* = 5–6). *p < 0.05, **p < 0.01, ***p < 0.001, ****p < 0.0001 by one-way ANOVA. Additional *p*-values not shown: *p* < 0.05 for vehicle vs fulvestrant; *p* < 0.0001 for vehicle vs every other treatment; *p* < 0.01 for fulvestrant vs palbociclib 35 mg/kg; *p* < 0.0001 for fulvestrant vs every other treatment. F, fulvestrant; L5 and L10, lasofoxifene at 5 and 10 mg/kg; P35 and P70, palbociclib at 35 and 70 mg/kg; SEM, standard error of the mean; V, vehicle

In vivo real-time luminescence data of Y537S mice showed that LAS alone at 5 or 10 mg/kg slowed tumor growth relative to vehicle, with a clear efficacy advantage over FUL (Fig. 3a). Inhibition was observed as early as 10 to 20 days after the treatment initiation with LAS or LAS/PAL combinations, unlike FUL alone, which did not have any effect at early time points. PAL alone at 35 mg/kg also suppressed primary tumor growth with a slightly slower increase over time than FUL, while the effect of 70 mg/kg PAL alone was similar to that of 10 mg/kg LAS. The combination of PAL with LAS or FUL enhanced the inhibitory activity of each drug alone, except for 5/35 mg/kg LAS/PAL versus LAS alone and 5 mg/mouse FUL/70 mg/kg PAL versus PAL alone. However, the LAS/PAL combinations were notably more effective than the FUL/PAL combinations based on the luminescence data.

Terminal tumor weights reflected the real-time luminescence imaging well. All treatments tested, including LAS, FUL, and PAL alone, and the combinations of PAL with LAS or FUL, significantly reduced primary tumor mass relative to vehicle control (Fig. 3b). Both doses of LAS alone significantly decreased tumor weights compared with FUL and PAL monotherapies (except for 10 mg/kg LAS vs 70 mg/kg PAL, *p* = NS). Interestingly, LAS reduced tumor weight more at 5 mg/kg than 10 mg/kg. While the effect of PAL was stronger at the higher dose tested, both doses of PAL were significantly more effective than FUL alone. Addition of PAL at either dose significantly improved the effectiveness of FUL, although the activity of FUL/PAL was not significantly different from that of PAL alone at corresponding doses. Moreover, the combinations of 5/70 mg/kg and 10/35 mg/kg LAS/PAL were significantly more effective than either drug alone. Compared with FUL/PAL, the LAS/PAL combinations led to significantly lower final tumor weight, except for 5/35 mg/kg LAS/PAL (*p* = NS).

### Effect of combination therapies on tumor metastasis

**Fig. 4:**
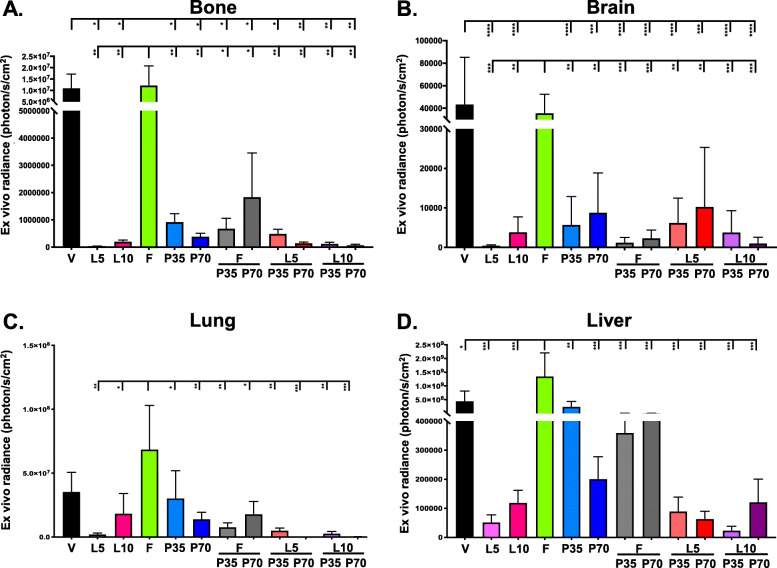
Effect of combination therapies on tumor metastasis in the Y537S ERα mutant model. Ex vivo radiance of luciferase activity was measured in excised **a** long bones (*n* = 10–12 legs), **b** brains (*n* = 5–6 mice), **c** lungs (*n* = 5–6 mice), and **d** livers (*n* = 5–6 mice). Error bars represent standard error of the mean. F, fulvestrant; L5 and L10, lasofoxifene at 5 and 10 mg/kg; P35 and P70, palbociclib at 35 and 70 mg/kg; V, vehicle. *p < 0.05, **p < 0.01, ***p < 0.001, ****p < 0.0001 by one-way ANOVA

Quantification of ex vivo radiance of excised long bones, brains, lungs, and livers from treated mice showed that monotherapies with both doses of LAS and PAL reduced metastases of Y537S mutant tumor cells to the four distal sites compared with vehicle only, while FUL alone did not show any effect (Fig. 4). Reduced metastases were generally observed with LAS versus PAL, and, similar to the effect on primary tumors (Fig. 3), with the lower versus higher dose of LAS at all four sites. The inhibitory effect of 5 mg/kg LAS was only enhanced by 70 mg/kg PAL at the lung (Fig. 4c). Improvements versus single agents on metastasis inhibition at the four sites were observed when 10 mg/kg LAS was combined with PAL, except for 10/35 mg/kg LAS/PAL at the brain (Fig. 4b) and 10/70 mg/kg LAS/PAL at the liver (Fig. 4d). While addition of PAL at either dose significantly improved the effectiveness of FUL, 70 mg/kg PAL alone was more beneficial than its combinations with FUL at the bone, lung, and liver (Fig. 4a, c, d). Notably, LAS/PAL combinations were in general more effective than FUL/PAL combinations at inhibiting metastasis of Y537S cells to all distal sites tested (Fig. 4a, c, d) except for the brain, for which only the 10/70 mg/kg LAS/PAL combination elicited a stronger inhibitory effect versus FUL/PAL (Fig. 4b).

**Fig. 5:**
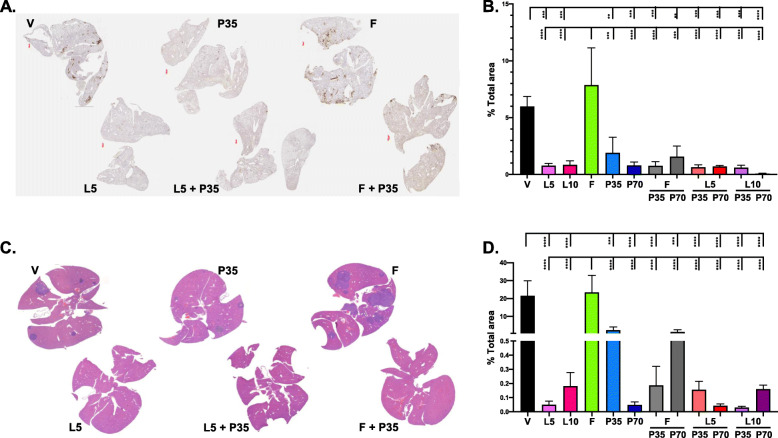
Histological analysis of lungs and livers in the Y537S ERα mutant model. **a** Representative IHC staining of lungs with anti-luciferase antibody showing lung metastases (brown). **b** Quantitation of the IHC staining showing metastasis as percent of total lung area (*n* = 5–6 mice). **c** Representative H&E staining of livers. **d** Quantitation of the H&E staining showing the area of tumor cells normalized to the total liver area (*n* = 5–6 mice). Error bars represent standard error of the mean. F, fulvestrant; L5 and L10, lasofoxifene at 5 and 10 mg/kg; P35 and P70, palbociclib at 35 and 70 mg/kg; V, vehicle. *p < 0.05, **p < 0.01, ***p < 0.001, ****p < 0.0001 by one-way ANOVA

Data from histological assessments of metastatic burden in the lung, liver, and bone were consistent with the ex vivo imaging results. LAS was substantially more effective than FUL in preventing metastasis to the lung and liver, regardless of the presence of PAL (Fig. 5). Compared with single agents alone, FUL plus 35 mg/kg PAL reduced metastatic coverage in both the liver and the lung (Fig. 5b, d), 10/70 mg/kg LAS/PAL did in the lung (Fig. 5b), and 10/35 mg/kg LAS/PAL did in the liver (Fig. 5d). H&E and IHC staining of bone marrow showed that 5 mg/kg LAS completely blocked tumor metastases to the bone, in contrast to vehicle control (Supplementary Figure S3). Both bone metastases and necrosis were observed in vehicle- and FUL-treated mice, but not in other treatment groups (Supplementary Table S2).

### Binding affinity for ERα LBD

Binding affinity of LAS for WT ERα LBD (*K*_i_ = 0.21 ± 0.06 nM) was comparable to that of E2 (*K*_d_ = 0.22 ± 0.11 nM), but lower compared to 4-OHT (*K*_i_ = 0.12 ± 0.003 nM) and FUL (*K*_i_ = 0.13 ± 0.03 nM) (Table 1). *ESR1* mutations reduced the affinities of LAS, 4-OHT, and FUL by approximately 10 to 40 folds relative to that for WT. The *K*_i_ of LAS, 4-OHT, and FUL was 2.34 ± 0.60 nM, 2.64 ± 0.40 nM, and 3.68 ± 0.77 nM, respectively, for Y537S binding, and 2.19 ± 0.24 nM, 2.29 ± 0.80 nM, and 5.06 ± 1.16 nM, respectively, for D538G binding (Table 1).

**Table 1:** Ligand-binding affinities for WT and mutant ERα LBDs

	K_**i**_±SD, nM (fold change over WT)		
Ligand	WT	Y537S	D538G
17β-Estradiol^a,b^	0.22±0.11	1.40±0.54 (6.36)	1.77±0.66 (8.05)
Lasofoxifene	0.21±0.06	2.34±0.60 (11.14)	2.19±0.24 (10.43)
4-Hydroxytamoxifen	0.12±0.003	2.64±0.40 (22.00)	2.29±0.80 (19.08)
Fulvestrant^b^	0.13±0.03	3.68±0.77 (28.31)	5.06±1.16 (38.92)

*LBD*, ligand binding domain; *SD*, standard deviation; *WT*, wild type

^a^Affinity was expressed as *K*_d_ (nM) for 17β-Estradiol

^b^Previously published data from Zhao et al. [14]

### Antagonist conformation of ERα LBD

**Fig. 6:**
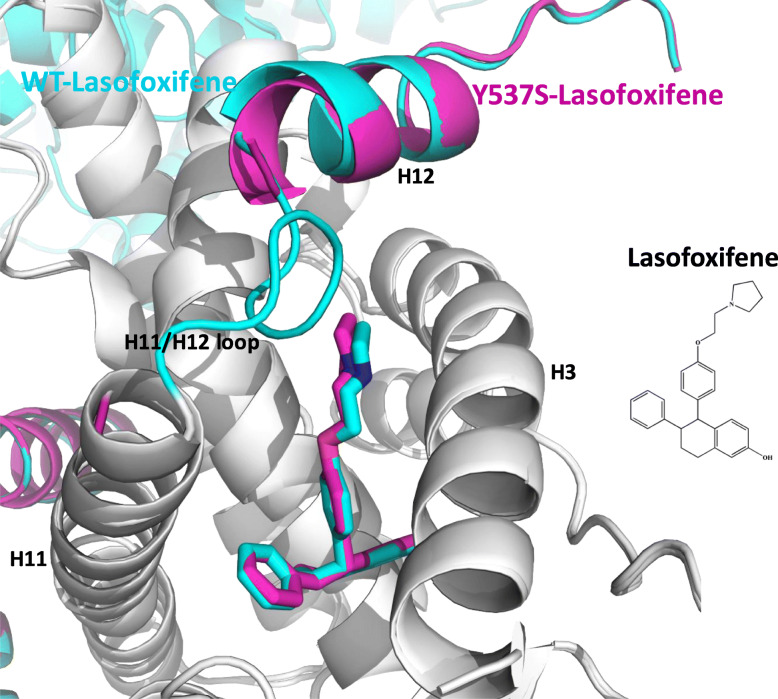
Merged x-ray crystal structures of WT (cyan) and Y537S (magenta) ERα LBDs in complex with lasofoxifene. PDBs: 6VJD and 6VGH

X-ray crystallography showed that LAS stabilized an antagonist conformation of both WT and Y537S ERα LBD, although the loop connecting H11 to H12 was absent in the Y537S structure (Fig. 6). Superposition of the crystal structures at alpha carbons shows that H12 is less helical and sits slightly away from AF2-cleft in the Y537S compared with to the WT. In the Y537S structure, LAS itself adopts a vector that places its pyrrolidine moiety closer to H12, likely to take the space that was occupied by the H11/12 loop in the WT structure. Only A chains were inspected as B chains show significant crystal contacts near H12.

## Discussion

We report for the first time the anti-tumor activity of LAS in mouse models of endocrine-resistant breast cancer. Luminescence imaging, although semi-quantitative, provided real-time data on tumor growth in vivo, which correlated well with final endpoint tumor weight measurements. The results demonstrate that LAS, a SERM without any demonstrable SERD activity, has clear advantages over FUL, the only clinically approved SERD, at inhibiting primary tumor growth and metastasis in ER+ breast cancer mouse xenografts expressing Y537S and D538G ERα mutants. Notably, LAS as a single agent was superior to both FUL and PAL alone in terms of tumor suppression and metastasis inhibition at the liver, lung, brain, and bone. While improvements were observed for both LAS/PAL and FUL/PAL versus single agent alone, LAS/PAL was significantly more effective than FUL/PAL, demonstrating the potential of LAS in controlling endocrine-resistant ER+ breast cancer.

A MIND model with tumor cells injected directly into the milk ducts was used to establish mouse xenografts. This model was originally developed to follow the natural progression of ductal carcinoma in situ (DCIS) and later used to study invasive breast cancer [28, 29]. Unlike traditional mammary fat pad xenografts which acquire basal characteristics, the MIND model preserves the luminal phenotype of the cells, thus closely mimicking the original ER+ tumor [29]. In addition, the Y537S and D538G *ESR1* mutations are heterozygous in the engineered mutant cell lines, more accurately reflecting the natural history of these mutations in refractory breast cancers [10] and adding to the clinical relevance of these in vivo models.

The improved responses of the ERα mutant tumor explants to LAS alone versus FUL alone are consistent with a published study in cell culture models, which showed that LAS retained its potency in Y537S and D538G ERα mutant cells relative to WT ERα cells, whereas potency for FUL was reduced in the mutant cells [23]. Additionally, the open-label, phase 2a, plasmaMATCH trial showed that FUL alone was ineffective in patients with advanced breast cancer and *ESR1* mutations [32], in agreement with our finding of low activities of FUL in *ESR1*-mutated models. The improved bioavailability of LAS relative to other SERMS, combined with its long half-life and extensive volume of distribution [20, 33], likely contributes to its high anti-tumor activity towards ERα mutant tumors. In contrast, the poor pharmaceutical properties of FUL and its potentially insufficient exposure in mutant tumors at the current doses could limit the activity of FUL [23, 34, 35].

To better understand the molecular basis of LAS potency against ERα mutants, biochemical studies were undertaken. Ligand binding assays showed that LAS, like 4-OHT and FUL, had reduced affinities to Y537S and D538G mutants, although the change was smaller compared with 4-OHT or FUL. The affinity data suggest that LAS binds to the ERα mutants as well as or slightly better than 4-OHT and FUL, which potentially promotes the antagonist activity of LAS. However, the difference in Y537S affinity for LAS versus FUL was not substantial enough to explain observed significant difference in anti-tumor activity between the two, considering the saturating dose of FUL used in our studies.

We also report x-ray crystal structures of WT and Y537S ERα LBDs bound to LAS, demonstrating that both form stable AF2 antagonist conformations. A comparison of the Y537S ERα LBD/LAS structure with that of WT ERα LBD/LAS complex revealed a disrupted H11/H12 loop by the side chain of LAS relative to WT LBD. This loop plays a key role in facilitating the constitutive activities of ERα with mutations in or near the N-terminus of H12 [36] and drugs that increase the dynamic character of H12 might improve the response of the mutants [13]. Moreover, the loop also contributes to ER stability and disruption of the loop can potentially lead to receptor degradation [13]. LAS has not been shown to affect WT ER degradation; however, its effect on Y537S and D538G mutant ERα needs further investigation. Additionally, x-ray crystal structures of SERMs and SERDs in complex with Y537S ERα LBD are also needed to uncover whether specific ligand-induced H12 conformations correlate with improved potencies in breast cancers harboring *ESR1* mutants. Overall, the loop disruption observed in Y537S LBD/LAS versus WT LBD/LAS could contribute to the improved efficacy of LAS.

Prevention of metastasis is essential for optimal treatment response and survival in women with therapy-resistant breast cancer. The Y537S ERα mutation is generally considered the most resistant to endocrine therapy among the known ERα LBD mutations [15]. In the current studies, Y537S tumors were found to be less sensitive to FUL treatment and more metastatic than D538G tumors. FUL was ineffective in reducing liver and lung metastases in the Y537S model, consistent with previous findings that FUL did not show improvement versus AIs in treating patients with visceral metastases [37]. LAS, on the other hand, significantly inhibited metastasis in vivo to the distal sites of lung, liver, bone, and brain, with almost complete blockades at 5 and 10 mg/kg, demonstrating clear advantages over FUL for controlling metastasis.

Improved survival with FUL/PAL versus FUL/placebo in metastatic ER+ breast cancer after endocrine failure was noted in the PALOMA-3 trial [24, 25]. A network meta-analysis showed that the combinations of CDK4/6 inhibitors, such as PAL, ribociclib, and abemaciclib, with high-dose FUL were among the most effective treatment options for advanced ER+ breast cancer as reflected in overall and progression-free survival [38]. In our studies, while the activity of FUL was significantly improved by PAL as expected, LAS/PAL was more effective than FUL/PAL in general, both at tumor suppression and metastasis inhibition in the Y537S model. Interestingly, LAS alone appeared to be as effective as the combination of LAS plus PAL in some cases. However, efficacies of these treatments need to be verified in patients. The antitumor activity of LAS versus FUL is currently being investigated in the phase 2 ELAINE study (NCT03781063) among women with locally advanced or metastatic ER+/HER2− breast cancer expressing ERα mutants. The follow-up ELAINEII study (NCT04432454) has recently started enrolling patients to further evaluate LAS plus abemaciclib. These studies will provide valuable clinical data on the efficacy and safety of LAS either as a monotherapy or in combination with a CDK4/6 inhibitor for treatment of *ESR1*-mutated advanced or metastatic breast cancer.

## Conclusions

In conclusion, when compared to FUL, LAS elicited greater anti-tumor activities in preclinical models of breast cancer expressing Y537S and D538G ERα. Its best-in-class pharmaceutical characteristics, likely contribute to observed differences between LAS and FUL. Importantly, LAS combined with PAL was in general more effective than the combinations of FUL with PAL in tumor and metastasis inhibition. These results have important clinical implications and demonstrate the potential of using LAS as an effective therapy for women with advanced or metastatic ER+ breast cancers expressing constitutively active *ESR1* mutations. The ongoing, phase 2 ELAINE studies in women with advanced or metastatic ER+/HER2− breast cancer expressing ERα mutants are expected to provide further clinical data regarding the efficacy and safety of LAS as an antitumor therapy.

## Supplementary Information

**Additional file 1: Supplemental Table S1.** X-ray crystallographic statistics. Description of data: The table summarizes X-ray crystallographic data collection and refinement statistics for ERα LBD WT/Lasofoxifene and ERα LBD Y537S/Lasofoxifene complexes.

**Additional file 2: Supplementary Table S2.** Summary of bone metastasis results (*n* = 5–6 mice). Description of data: The table summarizes the results on bone metastasis in different treatment groups.

**Additional file 3: Supplementary Figure S1.** 2mFo-DFc difference maps showing the maps for lasofoxifene in the (A) wild type and (B) Y537S ligand binding pocket of ERα ligand binding domain contoured to 1.5 σ. Description of data: The figure shows 2mFo-DFc difference maps for representative lasofoxifene ligands in the ERα LBD binding pocket for the wild-type and Y537S structures.

**Additional file 4: Supplementary Figure S2.** *In vivo* luminescence images of mice bearing tumors expressing MCF7 WT, Y537S, and D538G ERα at day 56 after treatment initiation. FUL, fulvestrant; LAS, lasofoxifene; Veh, vehicle. Description of data: The figure provides *in vivo* luminescence images of tumor-bearing mice after treatment with vehicle, fulvestrant, and lasofoxifene (3 mice per treatment group).

**Additional file 5: Supplementary Figure S3.** Histological analysis of excised long bones in the Y537S ERα mutant model. (A) An example of H&E staining of bone marrow for a vehicle-treated mouse. Insert shows bone metastases stained with anti-luciferase antibody (left) and H&E staining (right). (B) An example of H&E staining of bone marrow for a 5mg/kg lasofoxifene-treated mouse. No metastases were observed. Description of data: The figure shows examples of H&E and IHC staining images of bone marrow after treatment with vehicle and lasofoxifene.

## Data Availability

All data generated or analyzed during the current study are available from the corresponding author on reasonable request.

## Abbreviations

4-OHT: 4-Hydroxytamoxifen
AF2: Activating function-2
AI: Aromatase inhibitor
DCIS: Ductal carcinoma in situ
E2: Estradiol
ER: Estrogen receptor
FUL: Fulvestrant
GFP: Green fluorescent protein
H&E: Hematoxylin and eosin
IHC: Immunohistochemical
LAS: Lasofoxifene
LBD: Ligand binding domain
MBC: Metastatic breast cancer
MIND: Mammary intraductal
PDB: The Protein Data Bank
s.c.: Subcutaneous
SERD: Selective estrogen receptor down regulator
SERM: Selective estrogen receptor modulator
PAL: Palbociclib
PEARL: The Postmenopausal Evaluation and Risk-Reduction with Lasofoxifene trial
WT: Wild type
