# Supplementary material for: Lasofoxifene as a potential treatment for therapy-resistant ER-positive metastatic breast cancer
Source: Breast Cancer Res. 2021 May 12;23:54. doi: 10.1186/s13058-021-01431-w (PMC8117302; doi:10.1186/s13058-021-01431-w)
Supplement: Supplementary file 1 — Additional file 1: Supplemental Table S1. X-ray crystallographic statistics. Description of data: The table summarizes X-ray crystallographic data collection and refinement statistics for ERα LBD WT/Lasofoxifene and ERα LBD Y537S/Lasofoxifene complexes. [file 13058_2021_1431_MOESM1_ESM.docx]

**Supplemental Table S1. X-ray crystallographic statistics**

|  | **ERα LBD WT-Lasofoxifene** | **ERα LBD Y537S-Lasofoxifene** |
| --- | --- | --- |
| PDB ID | 6VJD | 6VGH |
| Data Collection | | |
| Space Group | P3_2_ | P6_5_ |
| a, b, c (Å) | 58.36, 58.36, 275.62 | 58.69, 58.69, 276.48 |
| α, β, γ (°) | 90.00, 90.00, 120.00 | 90.00, 90.00, 120.00 |
| Resolution Range (Å) | 29.18 – 1.80 | 28.71 – 2.10 |
| Number of Reflections (all/unique) | 89,624/20,842 | 71,296/23,765 |
| Completeness (Highest Resolution) | 91.8/86.9 | 90.9/81.2 |
| Redundancy | 4.3 | 3.0 |
| CC^1/2^ (Highest Resolution) | 0.607 | 0.749 |
| Refinement | | |
| R_work_/R_free_ | 17.1/21.0 | 21.6/27.4 |
| No. Atoms | 4,172 | 3,925 |
| Water Molecules | 763 | 256 |
| Ligand Molecules | 4 | 2 |
| Bond Lengths (Å) | 0.007 | 0.002 |
| Bond Angles (°) | 0.966 | 0.681 |
| Ramachandaran Plot Statistics | | |
| Preferred Number (%) | 99.16 | 98.87 |
| Additional Allowed (%) | 0.84 | 1.13 |
| Outliers (%) | 0 | 0 |

LBD, ligand binding domain; WT, wild type.
