# Supplementary material for: Lasofoxifene as a potential treatment for therapy-resistant ER-positive metastatic breast cancer
Source: Breast Cancer Res. 2021 May 12;23:54. doi: 10.1186/s13058-021-01431-w (PMC8117302; doi:10.1186/s13058-021-01431-w)
Supplement: Supplementary file 2 — Additional file 2: Supplementary Table S2. Summary of bone metastasis results (n = 5–6 mice). Description of data: The table summarizes the results on bone metastasis in different treatment groups. [file 13058_2021_1431_MOESM2_ESM.docx]

**Supplementary Table S2. Summary of bone metastasis results (n = 5–6 mice)**

|  | **Number of legs** | | |
| --- | --- | --- | --- |
| **Treatment** | **Total examined** | **Metastases**  **(% of bone marrow^a^)** | **Necrosis**  **(% necrosis)** |
| Veh | 12 | 3 (1%; 5%; 90%) | 1 (80%) |
| LAS 5 | 10 | none | none |
| LAS 10 | 10 | none | none |
| PAL 35 | 12 | none | none |
| PAL 70 | 12 | none | none |
| FUL | 10 | 2 (1%; 10%) | 1 (18%) |
| FUL + PAL 35 | 10 | none | none |
| FUL + PAL 70 | 10 | none | none |
| LAS 5 + PAL 35 | 12 | none | none |
| LAS 5 + PAL 70 | 12 | none | none |
| LAS 10 + PAL 35 | 12 | none | none |
| LAS 10 + PAL 70 | 12 | none | none |

FUL, fulvestrant; Las 5, lasofoxifene 5 mg/kg; Las 10, lasofoxifene 10 mg/kg; PAL 35, palbociclib 35 mg/kg; PAL 70, palbociclib 70 mg/kg; Veh, vehicle.

^a^Percentage was reported for each leg with metastasis.
