# Supplementary material for: Lasofoxifene as a potential treatment for therapy-resistant ER-positive metastatic breast cancer
Source: Breast Cancer Res. 2021 May 12;23:54. doi: 10.1186/s13058-021-01431-w (PMC8117302; doi:10.1186/s13058-021-01431-w)

**Supplementary Fig. S1:** 2mFo-DFc difference maps showing the maps for lasofoxifene in the (**A**) wild type and (**B**) Y537S ligand binding pocket of ERα ligand binding domain contoured to 1.5 σ.


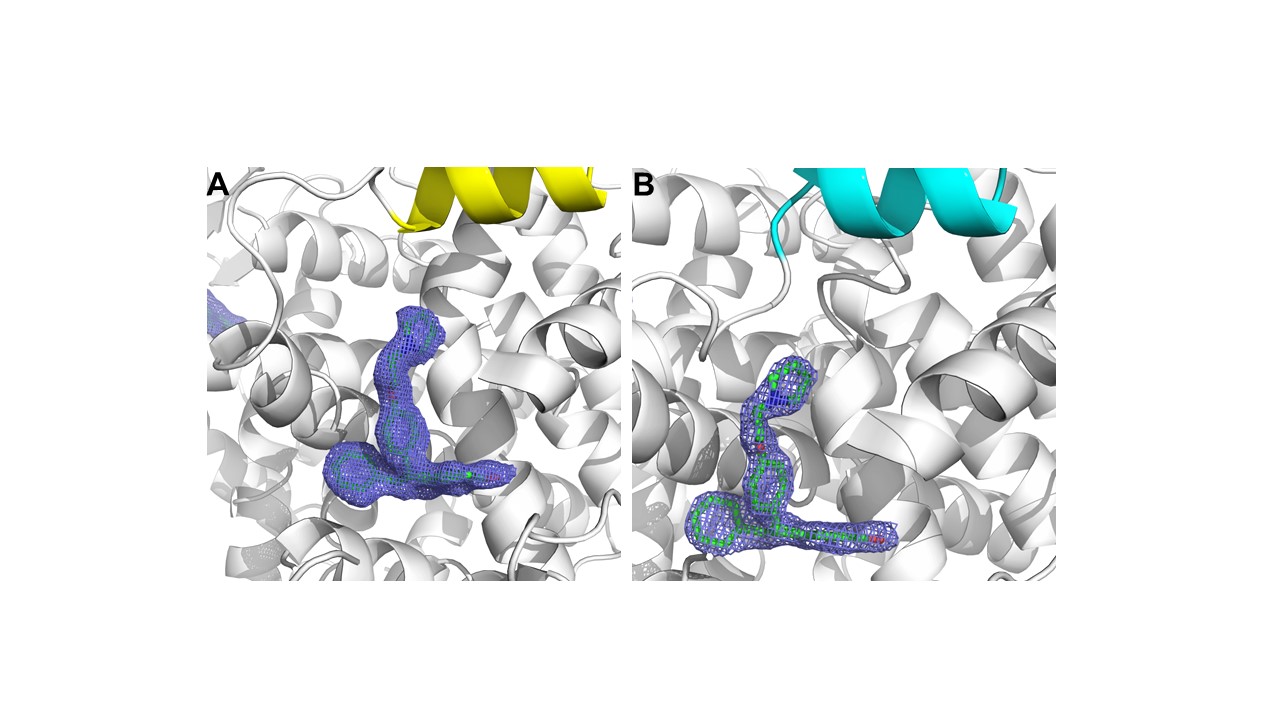

Supplement: Supplementary file 3 — Additional file 3: Supplementary Figure S1. 2mFo-DFc difference maps showing the maps for lasofoxifene in the (A) wild type and (B) Y537S ligand binding pocket of ERα ligand binding domain contoured to 1.5 σ. Description of data: The figure shows 2mFo-DFc difference maps for representative lasofoxifene ligands in the ERα LBD binding pocket for the wild-type and Y537S structures. [file 13058_2021_1431_MOESM3_ESM.docx]
