# Supplementary material for: Lasofoxifene as a potential treatment for therapy-resistant ER-positive metastatic breast cancer
Source: Breast Cancer Res. 2021 May 12;23:54. doi: 10.1186/s13058-021-01431-w (PMC8117302; doi:10.1186/s13058-021-01431-w)
Supplement: Supplementary file 4 — Additional file 4: Supplementary Figure S2. In vivo luminescence images of mice bearing tumors expressing MCF7 WT, Y537S, and D538G ERα at day 56 after treatment initiation. FUL, fulvestrant; LAS, lasofoxifene; Veh, vehicle. Description of data: The figure provides in vivo luminescence images of tumor-bearing mice after treatment with vehicle, fulvestrant, and lasofoxifene (3 mice per treatment group). [file 13058_2021_1431_MOESM4_ESM.docx]

**Supplementary Fig. S2:** *In vivo* luminescence images of mice bearing tumors expressing MCF7 WT, Y537S, and D538G ERα at day 56 after treatment initiation. FUL, fulvestrant; LAS, lasofoxifene; Veh, vehicle.
