# Supplementary material for: Lasofoxifene as a potential treatment for therapy-resistant ER-positive metastatic breast cancer
Source: Breast Cancer Res. 2021 May 12;23:54. doi: 10.1186/s13058-021-01431-w (PMC8117302; doi:10.1186/s13058-021-01431-w)
Supplement: Supplementary file 5 — Additional file 5: Supplementary Figure S3. Histological analysis of excised long bones in the Y537S ERα mutant model. (A) An example of H&E staining of bone marrow for a vehicle-treated mouse. Insert shows bone metastases stained with anti-luciferase antibody (left) and H&E staining (right). (B) An example of H&E staining of bone marrow for a 5mg/kg lasofoxifene-treated mouse. No metastases were observed. Description of data: The figure shows examples of H&E and IHC staining images of bone marrow after treatment with vehicle and lasofoxifene. [file 13058_2021_1431_MOESM5_ESM.docx]

**Supplementary Fig. S3:** Histological analysis of excised long bones in the Y537S ERα mutant model. (**A**) An example of H&E staining of bone marrow for a vehicle-treated mouse. Insert shows bone metastases stained with anti-luciferase antibody (left) and H&E staining (right). (**B**) An example of H&E staining of bone marrow for a 5mg/kg lasofoxifene-treated mouse. No metastases were observed.

**
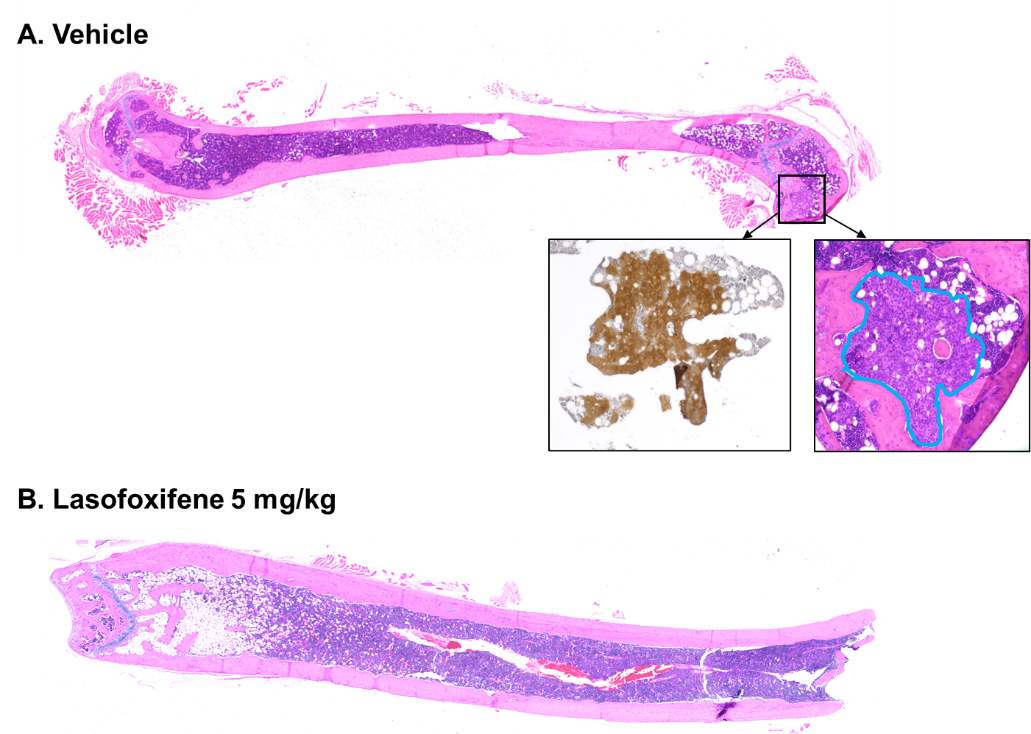
**
